# B-cell Lymphoma in retrieved femoral heads: a long term follow up

**DOI:** 10.1186/1471-2474-10-53

**Published:** 2009-05-20

**Authors:** Eline W Zwitser, Arthur de Gast, Mirjam JA Basie, Folkert J van Kemenade, Barend J van Royen

**Affiliations:** 1 Department of Orthopaedic Surgery, VU University Medical Center, Amsterdam, The Netherlands; 2 Department of Pathology, VU University Medical Center, Amsterdam, The Netherlands

## Abstract

**Background:**

A relatively high incidence of pathological conditions in retrieved femoral heads, including a group of patients having low grade B-cell lymphoma, has been described before. At short term follow up none of these patients with low-grade B-cell lymphoma showed evidence of systemic disease. However, the long term follow up of these patients is not known.

**Methods:**

From November 1994 up to and including December 2005 we screened all femoral heads removed at the time of primary total hip replacement histopathologically and included them in the bone banking protocol according to the guidelines of the American Associations of Tissue Banks (AATB) and the European Association of Musculo-Skeletal Transplantation (EAMST). We determined the percentage of B-cell lymphoma in all femoral heads and in the group that fulfilled all criteria of the bone banking protocol and report on the long-term follow-up.

**Results:**

Of 852 femoral heads fourteen (1.6%) were highly suspicious for low-grade B-cell lymphoma. Of these 852 femoral heads, 504 were eligible for bone transplantation according to the guidelines of the AATB and the EAMST. Six femoral heads of this group of 504 were highly suspicious for low-grade B-cell lymphoma (1.2%). At long term follow up two (0.2%) of all patients developed systemic malignant disease and one of them needed medical treatment for her condition.

**Conclusion:**

In routine histopathological screening we found variable numbers of low-grade B-cell lymphoma throughout the years, even in a group of femoral heads that were eligible for bone transplantation. Allogenic transmission of malignancy has not yet been reported on, but surviving viruses are proven to be transmissible. Therefore, we recommend the routine histopathological evaluation of all femoral heads removed at primary total hip arthroplasty as a tool for quality control, whether the femoral head is used for bone banking or not.

## Background

Allograft donor bone is successfully used in reconstructive tumor surgery and revision of total hip arthroplasties [1,2]. Bone banks collect femoral heads according to the safety procedures of the American Associations of Tissue Banks (AATB) and the European Association of Musculo-Skeletal Transplantation (EAMST) [3,4]. These constantly updated guidelines are of the utmost importance in order to prevent the transmission of infectious diseases.

Despite these extensive procedures, there have been recent reports of living cells within donor tissue after routine cryopreservation [5-7]. The potential risk of transmission of diseases from donor to recipient by these surviving cells remains uncertain, but there have been several reports on the development of infectious diseases due to the transplantation of contaminated allograft bone [8-14]. Previously we, and another group, recommended routine histological examination in the screening protocol because of a relatively high percentage of pathological conditions in retrieved femoral heads, including a group of patients having low grade B-cell lymphoma [15,16]. At short term follow up none of the patients with low-grade B-cell lymphoma in the femoral head showed evidence of systemic disease. However, the clinical consequences of these findings remained unknown. Therefore we continued our prospective study to screen histopathologically all retrieved femoral heads after total hip arthroplasty, including those that were not suitable for bone banking. We determined the percentage of B-cell lymphoma in all femoral heads, and in the group that met all criteria of the bone banking protocol, and will now report on the long-term follow-up.

## Methods

From November 1994 through December 2005 all femoral heads removed at the time of primary total hip replacement were collected according to the guidelines of the AATB and EAMST [3,4]. All patients gave written and signed informed consent. They were screened by a questionnaire covering their medical, social and sexual history. The questionnaire was based on pre-existing forms, followed the guidelines of the AATB and EAMST, and was written in the patient's native language. Additionally, the patients were interviewed by a doctor. A thorough physical and routine blood examination was performed. Blood was collected to determine the blood group, Rhesus factor, and white blood cell count. Screening tests were performed to exclude syphilis, HBV and hepatitis C virus (HCV), cytomegalovirus (CMV), HIV1 and 2 and HTLV type 1. An increased ESR was also used as a criterion for exclusion. All donors were retested for HIV1 and 2, syphilis, HBV, HCV and HTLV1 six months after the donation, taking into account the negative window period. After resection of the femoral heads, swabs from bone and capsule were taken for aerobic and anaerobic cultures. A core bone biopsy specimen (1 cm^3^) and a part of the capsule were retrieved for histopathological examination. Cores or biopsies were, in general, too big for electrolytical decalcification, as is routine in the laboratory for trephine biopsies. Thus they were decalcified in Kirstensen' solution in a daily controlled fashion. After decalcification, biopsies were routinely processed and microscopy was performed on Hematoxylin and Eosin. When required for more precise examination (i.e. lymphocytic collections that were monotoneous) additional stains and immunohistochemistry were performed to confirm diagnosis and, if possible, classified.

Grafts were stored at -80°C and six months later another blood examination followed. Earlier we described our bone bank screening procedure in detail [15]. Femoral heads of patients which were excluded somewhere in this bone banking selection process were sent for routine histopathological examination only.

Non-Hodgkin Lymphoma was diagnosed according to routine diagnostic procedures in the laboratory by expert hematopathologists. Criteria for diagnosis were an increase in lymphoid cells in general characterized by an interstitial pattern of infiltration, or paratrabecular localization of two or more nodules to packed marrow of cells with short chain restriction (kappa or Lambda) and uniform B-cell marker (CD20, CD79a, CD23 expression). In cases of concomitant elevated peripheral white blood cell counts, CLL was diagnosed.

We determined the percentage of low-grade B-cell lymphoma in all femoral heads and in the group that was suitable according to the bone banking protocol. Patients having low-grade B-cell lymphoma in the femoral head were referred to a hematologist-oncologist for further evaluation. Follow up of these patients consisted of clinical and radiological assessment on a yearly basis, review of hospital files and contact with family doctors. We assessed patients who had symptoms of systemic disease and who were treated by other physicians.

## Results

**Figure 1:**
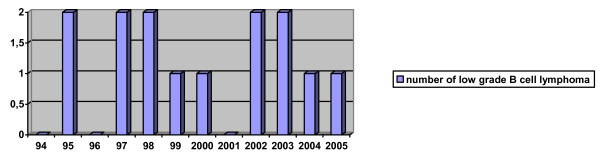
**Distribution of low grade B cell lymphoma found in retrieved femoral heads throughout the years in routine histopathological screening**.

From November 1994 to December 2005 we obtained 852 femoral heads removed at the time of primary total hip replacement from 737 patients (205 men, 532 women), mean age 69 years (Range 23 to 94 years). We reported earlier on the first 137 of these femoral heads [15]. Fourteen of 852 femoral heads were highly suspicious of low-grade B-cell lymphoma (1.6%). These fourteen femoral heads were retrieved from 13 donors. Mean age of these donors was 67 years (Range 51–79 years). Of 852 femoral heads, 504 fulfilled all criteria for donation according to the existing guidelines of EAMST and AATB. A total of 348 femoral heads were discarded because of a positive donor history (questionnaire), an increased ESR, or positive serological, histological or bacterial tests. They were also discarded if malignant disease was diagnosed within six months of donation. Some femoral heads have been rejected because of incomplete information from the screening tests. Six of 504 allografts fulfilling all criteria for donation were excluded based solely on the histopathological features of B-cell lymphoma (1.2%). These six femoral heads, retrieved from six different donors, were also excluded from clinical use and donation. We found variable numbers of low-grade B-cell lymphoma throughout the years. The distribution of low-grade B-cell lymphoma for each year is shown in figure 1. We performed long term follow up at a median of 7.2 years (Range 1–12 years). In this long term follow up one patient had developed a B-cell lymphoma in a lymph vessel in the inguinal region on the contralateral side, three years after the histopathological diagnosis of low-grade B-cell lymphoma was established. She underwent a lymph node excision and started with chemotherapy and radiotherapy. Currently this patient has been stable (non-progressive disease) for three years but continues to be monitored by a hematologist-oncologist. Another patient developed chronic lymphatic leukemia. No treatment was indicated but she is under the supervision of a hematologist-oncologist. Three other patients are still being monitored on a yearly base by a hematologist. They have yet to develop malignant disease.

## Discussion

In routine histopathological screening of 852 femoral heads, throughout the years we have found fourteen low-grade B-cell Non-Hodgkin Lymphoma (NHL) in thirteen patients. Even in the group of 504 femoral heads that were eligible for bone transplantation, six femoral heads in six patients were suspicious of low-grade B-cell lymphoma. We have performed a long term follow up of these thirteen patients in which we diagnosed low-grade B-cell NHL in their removed femoral heads. Two patients developed systemic malignant disease; one of them needed medical treatment for her condition. Three other patients are evaluated on a yearly basis by a hematologist.

Non-Hodgkin lymphoma (NHL) is a malignancy of the lymphatic system, caused by uncontrolled proliferation of B- or T-cell lymphocytes. It is relatively uncommon, but the standardized incidence has increased dramatically in the past few decades and is expected to increase by an additional 36.1% between 2000 and 2020 [17,18]. Extensive research to assess this increase shows a diversity of potential risk factors, such as diet, environmental and occupational factors, hair dye, viruses, hepatitis C infection, immunosuppression and blood transfusion. However, explanations accounting for all increases are not yet available [19-27]. Treatment of patients with NHL depends on histological type, clinical stage and prognostic group [28]. In low-grade B-cell lymphoma, by the time of diagnosis the disease is widespread and therefore incurable, but only a small group of these patients develops systemic malignant disease. Therefore a watch and wait approach is indicated. Palliative chemotherapy can be started at the time the patient presents with systemic manifestation of disease [29]. In our study one of the thirteen patients diagnosed with low-grade B-cell lymphoma developed systemic malignant disease for which she needed medical treatment. Four other patients are being monitored on a yearly basis by a hematologist.

Recent literature questions the necessity of routine histopathological examination of retrieved femoral heads. There are studies that find the cost-performance ratio of routine histopathological evaluation unfavorable because of the little clinical significance in changes of treatment from the diagnoses found [30-34]. Other studies recommend routine evaluation because of a recognized incidence of unsuspected, malignant diagnoses and as a tool for quality control [15,16,35]. The significance of these diagnoses is still unknown, but patients with neoplastic histopathological findings need monitoring and follow up. To our best knowledge, transmission of low grade B-cell lymphoma by bone donation has never been reported in medical literature. On the other hand, it has never been proven that bone with a (low grade) hematologous neoplasm is not transmissible by allogenic bone transplantation to an otherwise healthy recipient.

## Conclusion

In conclusion, we found variable numbers of low-grade B-cell lymphoma throughout the years in routine histopathological screening of femoral heads retrieved from patients treated by total hip arthroplasty, even in a group that was eligible for bone transplantation. Two patients developed systemic malignant disease; one of them needed medical treatment for her condition. Three other patients are still evaluated on a yearly basis by a hematologist. Therefore, we recommend the routine histopathological evaluation of all femoral heads removed during elective total hip arthroplasty as a tool for quality control, whether the femoral head is used for bone banking or not. In addition, all patients with neoplastic histopathological findings need monitoring and follow up.

## Competing interests

The authors declare that they have no competing interests.

## Authors' contributions

All authors have been involved in the development of the study design and research protocols. BJvR and AdG participated in the general coordination of the study and corrected the manuscript. EWZ and MJAB carried out data collection and drafted the manuscript. FJvK was responsible for the pathology. All authors read and corrected draft versions of the manuscript and approved the final manuscript.

## Pre-publication history

The pre-publication history for this paper can be accessed here:


